# Somatostatin Modulates Insulin-Degrading-Enzyme Metabolism: Implications for the Regulation of Microglia Activity in AD

**DOI:** 10.1371/journal.pone.0034376

**Published:** 2012-04-03

**Authors:** Grazia Tundo, Chiara Ciaccio, Diego Sbardella, Mariaserena Boraso, Barbara Viviani, Massimiliano Coletta, Stefano Marini

**Affiliations:** 1 Department of Experimental Medicine and Biochemical Sciences, University of Roma Tor Vergata, Roma, Italy; 2 Interuniversity Consortium for the Research on the Chemistry of Metals in Biological Systems, Bari, Italy; 3 Department of Pharmacological Sciences, University of Milan, Milan, Italy; Massachusetts General Hospital and Harvard Medical School, United States of America

## Abstract

The deposition of β-amyloid (Aβ) into senile plaques and the impairment of somatostatin-mediated neurotransmission are key pathological events in the onset of Alzheimer's disease (AD). Insulin-degrading-enzyme (IDE) is one of the main extracellular protease targeting Aβ, and thus it represents an interesting pharmacological target for AD therapy. We show that the active form of somatostatin-14 regulates IDE activity by affecting its expression and secretion in microglia cells. A similar effect can also be observed when adding octreotide. Following a previous observation where somatostatin directly interacts with IDE, here we demonstrate that somatostatin regulates Aβ catabolism by modulating IDE proteolytic activity in IDE gene-silencing experiments. As a whole, these data indicate the relevant role played by somatostatin and, potentially, by analogue octreotide, in preventing Aβ accumulation by partially restoring IDE activity.

## Introduction

The development of an efficient therapeutic strategy for the treatment of Alzheimer's disease (AD) requires a deeper understanding of the biochemical mechanism which leads to neuronal dysfunction and death [1]. Although molecular basis of AD remains largely unclear, there is considerable evidence which supports the so-called “amyloid cascade hypothesis”: AD may be viewed as a metabolic vicious cycle in which β-amyloid (Aβ) deposition into senile plaques drives neurodegeneration by triggering abnormal microglia activation, tau protein hyperphosphorylation and the consequent death of astrocytes and neurons. Aβ accumulation in the brain is the result of the imbalance between its biosynthesis and removal [2], [3]. Several proteases, such as insulin-degrading-enzyme (IDE), neprilysin (NEP) and matrix metalloproteinase 9 (MMP-9) are involved in Aβ degradation [4]–[6]. In this regard, a decrease in Aβ-degrading enzymes expression or activity, as a result of genetic factors, age and environmental conditions, may be a crucial aspect in AD pathogenesis [7]. Notably, IDE is a zinc metalloendopeptidase that hydrolyzes a wide range of substrates, including insulin, amylin, glucagon, atrial natriuretic factor and insulin-like growth factors I and II [8]. Even though its role in the onset of AD is not yet to be completely understood [5], the relevance of IDE in AD pathogenesis has been validated in works which have mapped the IDE gene on chromosome 10 [9], [10], making it a candidate gene for the AD-6 locus. Furthermore, it has been shown that, during aging, IDE decreases in the brain of AD patients [11]–[13]. Additionally, several studies underline a link between IDE haplotypes and SNPs in the IDE gene, associating them to AD [14]–[16]. We have recently demonstrated that somatostatin (sst) is a substrate and an allosteric modulator of IDE activity, which enhances the proteolytic processing of a synthetic Aβ-peptide [17]. Somatostatin depletion in the cortex and hippocampus of AD patients is directly connected to memory and learning impairment [18]–[20] and a strong reduction of sst-expressing neurons in the mouse CA1 hippocampal region seems to be associated to the onset of AD [21]. Somatostatin seems to be also involved in the regulation of neprilysin activity by affecting its expression and its synaptic localization [22]. Although molecular mechanisms coupling somatostatin depletion and AD remain unclear, somatostatin transmitter replacement has been identified as a potential pharmacological strategy for AD prevention [23], [24]. Furthermore, the activation of somatostatin neurotransmission by sst-releasing agents, such as FK960 and FK962, has been shown to significantly improve cognitive performances in animal models [25], [26]. On the other hand, although in the past, the use of the somatostatin analogue octreotide in clinical trials has not been unequivocally associated to the restoration of cognitive functions, there is some evidence which seems to demonstrate its potential therapeutic value [24], [27]. Since microglia secretes Aβ and IDE [28], [29] and displays three functionally active receptor subtypes for somatostatin (sst) (*i.e.* Receptors type 2, 3 and 4) [30], [31], we investigated sst effect on IDE expression in microglia cells. In this work, we show that in activated microglia, somatostatin regulates IDE expression, secretion and proteolytic activity toward Aβ, which suggests that somatostatin pathological depletion could be one of the key events leading to Aβ deposition.

## Materials and Methods

### Materials

Mouse BV-2 microglial cells were kindly provided by Prof. D. Rosato (University of Perugia, Italy) and N9 cells by Prof. R. Ciccarelli (University of Chieti, Italy) [32], [33]. Octreotide was a generous gift from Italfarmaco. RPMI 1640, fetal bovine serum, L-glutammine, penicillin, streptomycin, sodium pyruvate and ciprofloxacin were purchased from Euro Clone (Life Sciences Division, Italy). Minimum essential Eagle's medium, fetal calf serum, trypsin 2.5%, DNAse 1%, glucose, streptomycin, penicillin, L-glutammine, L-leucine methyl ester were obtained from Sigma-Aldrich (St. Louis, MO, USA). Human somatostatin was obtained from Sigma-Aldrich (St. Louis, MO, USA); recombinant insulin degrading-enzyme was purchased from Calbiochem (San Diego, CA, USA); β-amyloid (1–40) was obtained from Anaspec (San Jose, CA, USA). An IDE siRNA SmartPool and a non-specific control pool were synthesized by Dharmacon (Lafayette, CO, USA). Each pool contains four individual siRNA duplex sequences. Lipofectamine and OptiMEM medium were obtained from Invitrogen (Carlsbad, CA, USA). A β-amyloid(1–40) Elisa kit was obtained from IBL-Humburg (Hamburg, Germany); an RNasy Plus micro kit for RNA isolation was obtained from Qiagen (Hilden, Germany) and a Masterscript kit and SYBER ROX Master Mix came from 5PRIME (Humburg, Germany). All other chemicals were from Sigma-Aldrich.

## Methods

**Cell culture.** BV-2, a murine microglial cell line, was cultured in RPMI 1640 supplemented with 15% fetal bovine serum (FBS), L-glutammine (2 mM), penicillin (50 IU/ml), streptomycin (50 µg/ml), sodium pyruvate (1 mM) and ciprofloxacin (0.03 mM). Cells were seeded on 96-well plates (1×10^4^/well) and grown overnight at 37°C in 5% CO_2_ [32].

**Astrocytes and microglia cell cultures.** Primary cultures of glial cells were prepared from 2-day-old newborn rats (Sprague–Dawley, Charles River, Calco, Italy). Cerebral hemispheres were freed of the meninges and were mechanically disrupted. Cells were dissociated in a solution of trypsin 2.5% and DNAse 1%, filtered through a 100-µm nylon mesh, and plated in a 75 cm^2^ flask (5×10^6^/flask) in minimum essential Eagle's medium (MEM) supplemented with 10% fetal calf serum, glucose (0.6%), streptomycin (0.1 mg/ml), penicillin (100 IU/ml) and L-glutammine (2 mM). Glial cultures were fed twice a week and grown at 37°C in a humidified incubator with 5% CO_2_. A layer of astrocytic cells was obtained through the vigorous shaking of a confluent 10-day-old monolayer of mixed glial cells [34]. Cultures of enriched astroglia were treated further with 5 mM L-leucine methyl ester to eliminate microglia (97% homogeneity). Isolated astroglial preparations were then seeded in 96-well plates (6×10^4^/well) in MEM with supplements as above. Microglia were isolated by shaking glial cultures at 260 rpm for 2 hrs. Microglia which dislodged into the medium were purified by plating for 30 min in 96-well plates (6×10^4^/well). Contaminating cells were removed with supernatant. These conditions allowed us to obtain highly enriched microglial cultures with 98% homogeneity, as assessed by immunocytochemistry with *Griffonia simplicifolia* isolectin B4.

All animal protocols have been performed to Department of Pharmacological Sciences, University of Milan. The experiments were designed so as to minimize the number of animals used. Animal protocols for these studies have been approved for Department of Pharmacological Sciences by the Ministry of Health and were conducted with minimal suffering of the animals and strictly following European Community (ECC Directive no. 609/86) and local (Italian legislative Decree no. 116, January 27, 1992) regulations for animal care. All animal care procedures were in accordance with the local Animal Care Committee, and no weight loss or death was observed after we had received them in our animal facility.

**Treatment and sample preparation.** Indicated concentrations of somatostatin (dissolved in 0.1 M phosphate buffer pH 7.3) were added to the cell culture medium on the day after plating. Stimulation was carried out overnight at 37°C in 5% CO_2_. Supernatants of stimulated and nonstimulated cells were collected and frozen at −20°C until use, while the monolayers were lysed in a solubilization buffer containing 50 mM NaCl, 1% Triton, 50 mM Tris-HCl, 1 mM phenylmethylsulfonylfluoride in the presence of a protease inhibitor cocktail with broad specificity for the inhibtion of serine, cysteine, aspartic and aminopeptidases (from Sigma-Aldrich).

**Qualitative Insulin-degrading-enzyme analysis by Western Blotting.** Total protein concentration of cell lysates was determined by a Bradford assay [35]. 10 µg of each sample were separated on a 10% SDS-PAGE gel and transferred on Hybond-ECL nitrocellulose filters (Amersham Biosciences, Piscataway, NJ, USA) for 1 h at 4°C. Membranes were blocked in a PBS Tween (T-PBS) 0.01%, 5% fat-free milk solution, then probed with IDE rabbit polyclonal antibody BC2 (Covance, Princeton, NJ, USA, 1∶3000 in T-PBS-0.1% and incubated with an Horse Radish Peroxidase-conjugated anti-rabbit IgG antibody (Biorad, Hercules, CA, USA, 1∶50000 in T-PBS 0.2% fatty free milk). Immunoreactive signals were detected with an ECL Advance Western Blotting Detection Kit (Amersham Biosciences). Densitometric analysis was performed by Image Quant TL program.

**Secreted IDE quantification in BV-2 cell supernatants by ELISA.** IDE goat polyclonal antibody K-20 (Santa Cruz Biotechnology, Santa Cruz, CA, USA) was diluted in PBS 1× and coated on a microtiter plate at a final concentration of 0.5 µg/ml. Unsaturated binding sites were blocked in T-PBS-0.01% 5% fat-free milk solution before exposure to BV-2 cell supernatants. An IDE rabbit polyclonal antibody BC2 (1∶3000in T-PBS 0.01%) and a Horse Radish Peroxidase-conjugated anti-rabbit IgG antibody (1∶50000 in T-PBS 0.2% fat-free milk) were used for IDE quantification. IDE concentration was determined by using a TMB colorimetric kit according to product instructions. The absorbance was measured at 450 nm in a microtiter plate reader (Tecan, Switzerland). A standard curve was performed by using known concentrations of recombinant IDE (Calbiochem).

**Quantitative real-time PCR.** The total RNA from each sample of BV-2 cells was isolated using an RNeasy Plus micro kit for RNA isolation according to the manufacturer's instructions. cDNA of total RNA was generated in two reaction volumes of Masterscript kit using random hexamers. Real-time PCR was performed by SDS7700 (Applied Biosystem, Foster City, CA, USA) with Manual SYBER ROX Master Mix. GAPDH was measured as the internal control. Primer sequences were: ACGAGGCTATACGTCCAAGATTG and ATTGCCACCCGCACATTTT (Forward and Reverse, respectively) for IDE; AACTTTGGCATTGTGGAAGG and CACATTGGGGGTAGGAACAC (Forward and Reverse, respectively) for GAPDH. The following cycles were performed: initial denaturation cycle at 95°C for 2 min, followed by 40 amplification cycles at 95°C for 15 s, at 58°C for 30 s and at 68°C for 30 s.

**Analysis of MMP-9 in Zymography and Western Blotting.** Aliquots of supernatants from cell culture samples were run under non-reducing denaturant conditions on 10% polyacrylamide gels containing 1 mg/mL gelatine (Merck, Darmstadt, Germany), soaked twice in 2.5% Triton X-100 for 20 min and then incubated for 18 hrs in the developing buffer (20 mM Tris, 5 mM CaCl_2_, pH 7.4). For visualization purposes, the gels were stained with Coomassie Brilliant Blue R-250 and destained with 10% acetic acid and 20% methanol solution. For the Western blotting analysis of MMP-9 in cell lysates, 10 µg of whole cell lysate were separated on 10% polyacrylamide gel and then transferred onto a Hybond nitrocellulose membrane. Non-specific sites were blocked with a 0.025% T-PBS 5% fat-free milk solution and filters were then probed with a polyclonal anti-MMP-9 antibody (Sigma Aldrich, St. Louis, CO 1∶2000 in 0.1% T-PBS) and thereafter with a Horse Radish Peroxidase-conjugated anti-rabbit IgG antibody, (1∶50000 in T-PBS 0.2% fat-free milk). Immunoreactive signals were detected with an ECL Advance Western Blotting Detection Kit (Amersham Biosciences).

**IDE siRNA Transfection.** BV-2 cells were used for transfection when 30%–40% confluent in order to obtain the greatest transfection efficiency. Cells were transfected with 20 pmol of IDE-siRNA or siRNA control pool using Lipofectamine in OptiMEM media according to the manufacturer's recommendations. Somatostatin stimulation was performed 48 h after transfection.

**ß-amyloid(1–40) quantification in BV-2 conditioned medium with sandwich ELISA.** Human somatostatin was added at different concentrations to the culture medium of control and IDE-siRNA transfected BV-2 cells. According to the manufacturer's instructions, the incubation was carried out for 1 h at 37°C and the supernatants collected for β-amyloid(1–40) quantification by ELISA. Equal volumes of each sample were incubated overnight at 4°C on plates pre-coated with anti-Aβ (35–40) Mouse IgG. HRP conjugated anti-mouse Aβ(1–16) rabbit IgG was incubated at 4°C for 1 h. Reactivity was developed with TMB for 30 min at room temperature and stopped with 1 N H_2_SO_4_. The absorbance was measured at 450 nm with a microtiter plate reader. A standard curve was obtained with Aβ(1–40).

**Statistical analysis.** One-way analysis of variance (ANOVA) was used to determine significant differences among groups. Tukey's honestly significant difference post hoc test was used for pair wise comparisons after the analysis of variance.

## Results

### Somatostatin modulation of IDE expression

**Figure 1:**
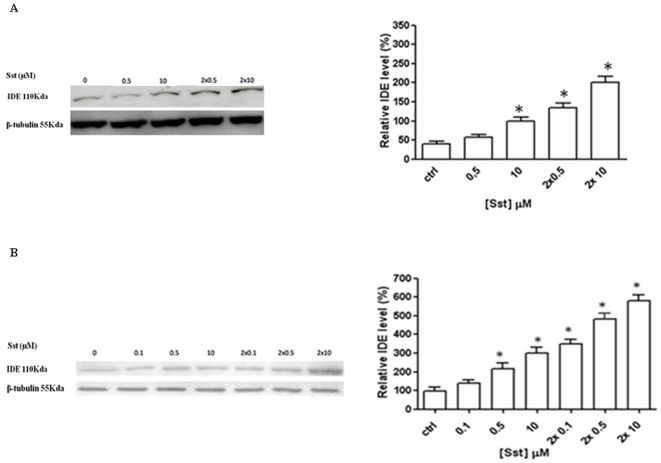
Somatostatin induces an increase of IDE expression in microglia cells. Western blot analysis of normalized lysis samples from rat primary microglia (A) and BV-2 (B) indicates that IDE level increases after 24 hrs of somatostatin incubation, while the internal control ß-tubulin is constant. The additional incubation with sst after 6 hrs from first round strengthens the effect on IDE expression (left panel). Densitometric analysis of IDE WB signals, average ± ES of 5 independent experiments in triplicate (right panel). *P<0.05, one-way ANOVA, followed by Tukey's test, n = 15.

BV-2 cells as well as primary rat microglia were stimulated with different sst concentrations and the IDE level was analyzed by Western blotting in cell lysates collected after 24 hrs of incubation. As shown in Figure 1, sst increases IDE expression in primary (A) and BV-2 cells (B). Since sst half life in the serum is short [36], the same sst concentration was re-added 6 hrs after the first administration. This addition further amplifies the positive modulation of IDE expression (Figure 1). It is interesting to note that WB analysis on cell lysates of sst-stimulated N9 microglia cell line confirms the enhancing effect on IDE expression (data not shown). In order to assess whether the effect of sst on IDE expression is a microglia characteristic, rat astrocytes which display three sst receptor subtypes as well (SSTR-1, SSTR-2, and SSTR-4) [37] were incubated with the same somatostatin concentrations (Figure S1). IDE level was analyzed by Western blotting in cell lysates collected after 24 hrs of sst incubation and as indicated in Figure S1, no detectable modulation of IDE expression was observed.

**Figure 2:**
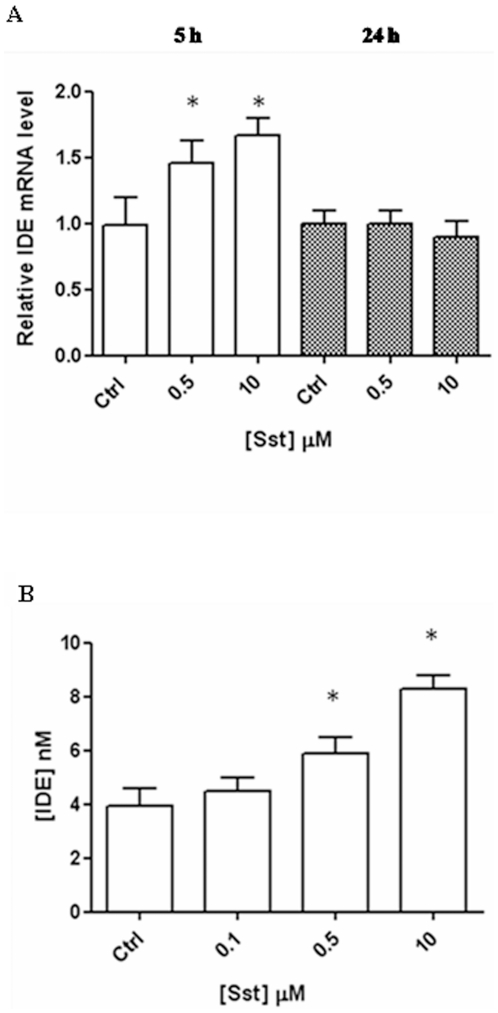
Somatostatin modulation on IDE level in BV-2 cells. (A) IDE mRNA increases after 5 hrs of incubation with somatostatin (white columns); 24 hrs after incubation, the level is similar to the control (grey columns). Basal mRNA levels were measured by real time PCR in individual preparations of BV2. Data were first normalized against GAPDH and then expressed setting the value measured in controls at 1. The results presented are the means ± ES (B) Elisa analysis of conditioned medium indicates that IDE level increases in BV-2 cells after 24 hrs of incubation as a function of somatostatin concentration. The results presented are the means ± SEs of five independent experiments in triplicate. P<0.05, one-way ANOVA, followed by Tukey's test, n = 15.

Since there was no principle difference in the response to somatostatin in microglia cell lines compared to primary rat microglia, immortalized BV-2 cells were subsequently used. The sst-mediated up-regulation of IDE in BV-2 cells was further checked by quantifying the mRNA using a RealTime-PCR. A time-course investigation reveals that IDE mRNA increases linearly with the sst concentration within 5 hrs to incubation and turns back after 24 hrs, suggesting a transcriptional effect triggered by sst in BV2 cells (Figure 2A).

### Somatostatin effect on IDE secretion

It is known that a small fraction of IDE (ranging from 3% to 10%) is secreted into the extracellular space, where it presumably interacts with its substrates, such as insulin and β-amyloid [8], [38]. In order to determine whether, besides inducing synthesis, sst also stimulates IDE secretion, the enzyme concentration in cell supernatants was quantified 24 hrs after sst administration. As reported in Figure 2B, the neuropeptide induces the increase of IDE secretion in a similar way to WB and RT-PCR (Figue 1B and 2A). This result indicates that somatostatin acts not only by modulating IDE expression, but also by affecting IDE secretion and increasing the amount of the enzyme in the extracellular space.

### Somatostatin effect on MMP-9 activity

**Figure 3:**
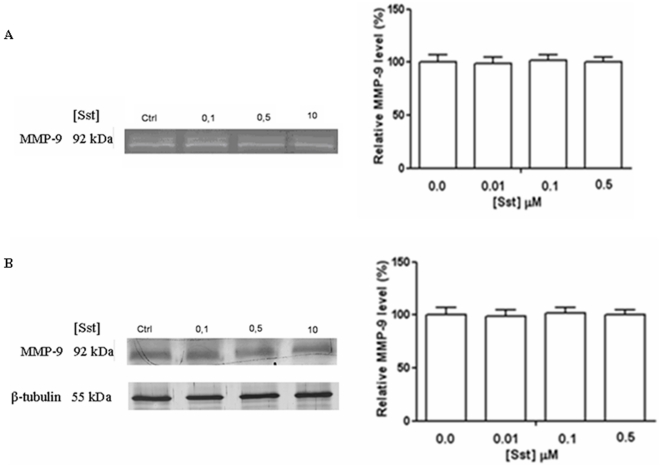
Effect of somatostatin on MMP-9 expression. (A) Zymographyc analysis of cell culture supernatants harvested after 24 hrs of incubation indicate that somatostain administration does not modulate gelatinolytic activity of MMP-9 in each of the experimental conditions. (B) In Western blotting analysis of cell lysates a polyclonal anti-MMP-9 antibody recognizes a single band corresponding to the pro-MMP-9 (92 kDa) which is not modulated over each experimental condition. Analysis of β-tubulin represents the internal control. The results presented are the means ± ES of three indipendent experiments in triplicate, n = 9.

In addition to their ability to phagocyte β-amyloid, microglia can also clear Aβ by degradation through the production of Aβ-degrading enzymes, such as IDE, neprilysin and MMP-9 [29], [33], [39]. Therefore, we assessed whether sst is involved in the regulation of MMP-9 level. For this purpose, BV-2 cells were stimulated with different sst concentrations and MMP-9 activity was measured by zymographyc analysis (Figure 3A). No detectable modulation of MMP-9 gelatinolytic activity was observed for each experimental condition. Qualitative analysis of MMP-9 content was further performed on total cell lysates. A polyclonal anti-MMP-9 antibody recognizes a single band around 92 kDa, corresponding to the pro-MMP-9, which is constant over the different sst concentrations (Figure 3B). This result rules out any involvement of sst in the modulation of MMP-9 expression, secretion and activity.

### Octreotide modulation of IDE expression

**Figure 4:**
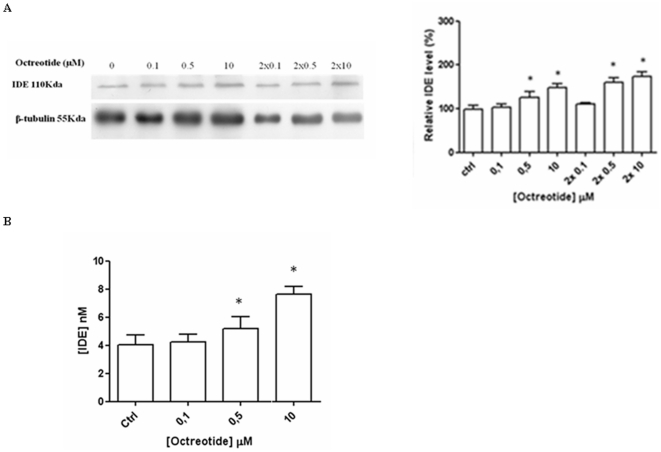
Somatostatin analogue octreotide increases IDE expression and secretion. (A) WB (left panel) and densitometric analysis of IDE (right panel) after 24 hrs incubation with the indicated concentrations of octreotide. (B) ELISA analysis on BV-2 medium after octreotide incubation reveals that the sst analogue induces IDE secretion. In every case, the results presented are the means ± ES of four independent experiments in triplicate. * P<0.05, oneway ANOVA, followed by Tukey's test, n = 12.

Recently, somatostatin transmitter replacement has been viewed as a potential AD therapeutical strategy [24], [40]. In this regard, BV-2 cells were stimulated with octreotide, a long-acting octapeptide somatostatin analogue commonly used in clinical trials, in order to verify the effect on the expression pattern of IDE [41]. 0.1–10 µM octreotide induces a dose-dependent increase of IDE expression (Figure 4A) in a similar way to what can be observed in the presence of somatostatin (Figure 1). A similar result can also be obtained for IDE secretion, by using an ELISA assay on BV-2 cell supernatants collected 24 hrs after the administration (Figure 4B). Therefore, these findings indicate that octreotide and sst exert a very similar effect on IDE expression and secretion in BV-2 cells (see Figures 1, 2 and 4).

### Somatostatin regulation of Aβ(1–40) degradation by insulin-degrading-enzyme

**Figure 5:**
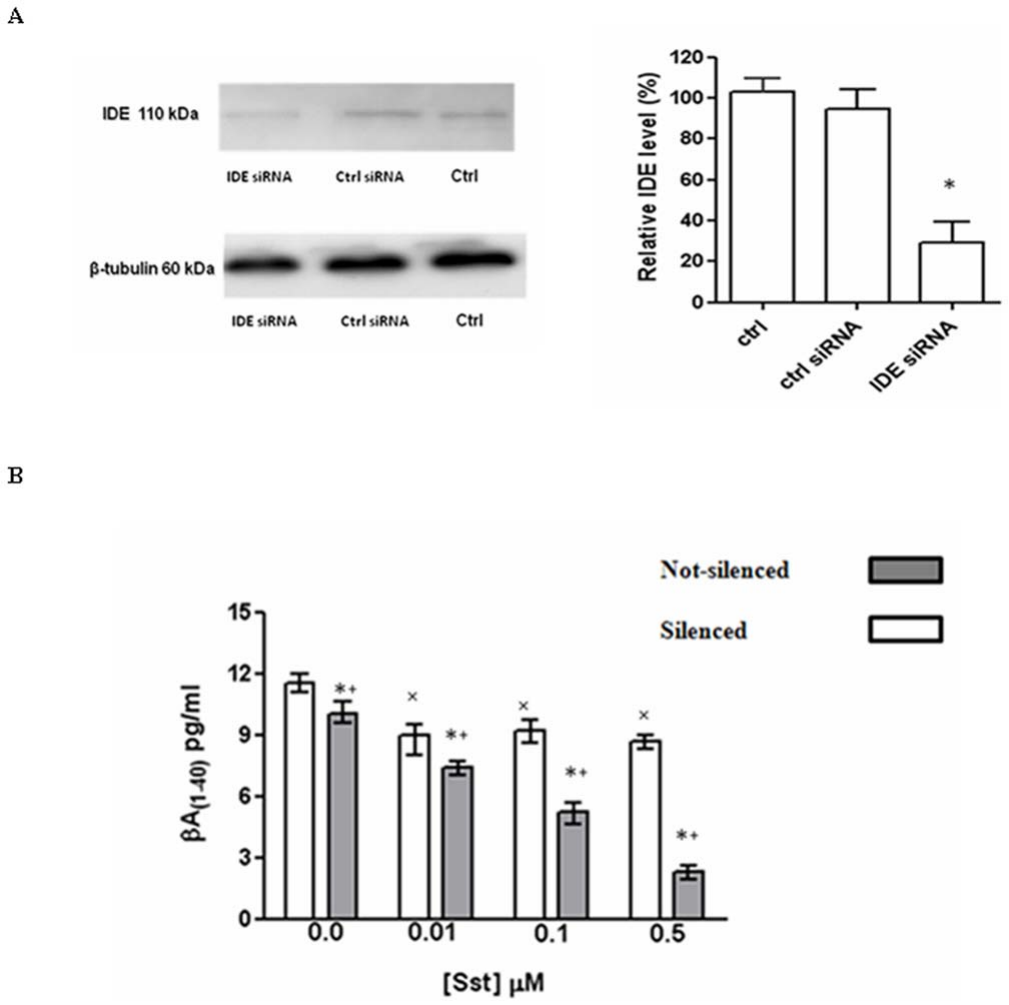
Somatostatin regulates IDE activity enhancing IDE-dependent Aβ degradation. (A) BV-2 cells transfected with a specific IDE siRNA pool show reduced levels of IDE protein, compared to cells transfected with a nonspecific control siRNA pool (right panel). (B) Aβ(1–40) quantification through sandwich ELISA reveals that the levels of Aβ are drastically reduced in the presence of IDE steady level (grey columns) compared to IDE-silencing samples (white columns). The results presented are the means ± ES of three independent experiments in triplicate. P<0.05, one-way ANOVA, followed by Tukey's test, n = 9. *Significantly different from internal control. ^+^Significantly different from silenced sample in the absence of somatostatin. ^×^Significantly different from not-silenced sample in the absence of somatostatin.

We have previously shown that somatostatin is an IDE substrate and an allosteric modulator of recombinant IDE enzymatic activity toward a fluorogenic β-peptide [17]. Therefore, we aimed to verify whether sst by allosteric modulation of endogenous IDE could affect the clearance of the Aβ peptide secreted by BV-2 cells. To this purpose, IDE expression was silenced through a siRNA-based approach. At the maximum rate of IDE silencing (corresponding to about 60%–70%, see Figure 5A), different somatostatin concentrations (ranging between 0.01 µM and 0.5 µM) were added to the culture medium of both silenced and non-silenced cells. Aβ(1–40) was quantified in cell supernatants after 1 h of somatostatin stimulation, a time interval during which there was no accumulation of newly synthesized IDE, a fact that was demonstrated through Western Blotting analysis on cell lysates (data not shown). Aβ(1–40) concentration was slightly reduced at sst 0.01 µM in both silenced and not-silenced cells. In spite of this, Aβ(1–40) concentration was not further altered in the medium of silenced cells by increasing sst concentrations, while it was progressively reduced in the medium of non-silenced cells (Figure 5B), suggesting that the reduction of Aβ observed at sst 0.01 in silenced cells could be due to the effect of sst on residual IDE. Therefore, at physiological concentrations, sst modulates IDE activity also by promoting Aβ(1–40) degradation in the BV-2 cells supernatants.

## Discussion

Although several pathological mechanisms of AD progression have been described, ranging from protein aggregation to oxidative stress, mitochondrial failure, metal dyshomeostasis and microglia dysfunction, the important role of amyloid deposition is now widely recognized [7]. In this regard, the extracellular Aβ degrading enzymes (such as IDE, MMP-9 and NEP) are now considered promising therapeutic targets for AD treatment [33]. Notably, IDE is the main extracellular protease secreted by microglia involved in Aβ degradation, even though the molecular basis of IDE regulation are poorly known [5]. Microglia seems to cover a two-fold role in AD pathogenesis. Firstly, early microglia activation exerts a neuroprotective effect by promoting Aβ clearance. Secondly, as the disease progresses, the microglia Aβ-clearing capability is compromised [38], [41]. This downregulation is followed by an increase of Aβ released by neurons and by a worsening of the disease [32]. We previously demonstrated that sst is an allosteric modulator of IDE [17]. In this work, we further study the effect of sst on IDE showing that the neuropeptide somatostatin also specifically regulates IDE, the main extracellular Aβ protease, by affecting its expression and secretion in both primary and BV-2 microglia cells. Somatostatin triggers IDE gene transcription and protein which displays a different turnover rate: IDE-mRNA reaches a maximum of transcription within 5 hrs after stimulation, returning to the basal level within 24 hrs, whereas the protein concentration increases in cell lysates and supernatants at later times, being still clearly evident after 24 hrs. Through this pathway, somatostatin enhances IDE secretion, strengthening the pool of active enzymes which interact with β-amyloid and other IDE extracellular substrates. This effect is specific for IDE since sst does not affect either secretion and activity of MMP-9, another enzyme which is active in Aβ degradation. It is known that IDE and somatostatin levels are altered in AD progression [3], [20]. It is thus conceivable that sst depletion results in a decrease of IDE expression and secretion contributing to the pathological deposition of β-amyloid in the brain. In addition, since the Aβ chronic accumulation triggers a further reduction in sst level [18], all these events could represent a vicious cycle which ultimately favors Aβ plaque formations. As matter of fact, somatostatinergic transmitter replacement is a potentially viable strategy in the treatment of AD, even though the pharmacological restoration of this deficit has not been unequivocally associated to a recovery of normal cognitive performances [24], [26], [40]. In this framework, we tested the octreotide effect on IDE expression, a somatostatin analogue currently used in the treatment of acromegaly, pituitary adenomes and pancreatic tumors [41], [42]. Here, we show that, like somatostatin, octreotide increases IDE expression and secretion, although the effects are generally reduced compared to the endogenous modulator. Somatostatin binds all five receptor subtypes with high affinity, whereas octreotide is a selective agonist, binding to receptor subtypes 2 and 5 with high affinity (but lower than somatostatin) and with moderate affinity to subtype 3 [41]. Therefore, this evidence might explain the discrepancy between sst and its analogue concerning the positive modulation of IDE expression, even though additional factors cannot be ruled out. Interestingly, we also observed that in astrocytes, incubation with somatostatin does not have any significant detectable effect of IDE expression, reinforcing the physiological relevance of somatostatin action on IDE secretion in microglia cells. This discrepancy is probably due to the different expression pattern of sst receptor on microglia (*i.e.* SSTR-2, SSTR-3, and SSTR-4) and astrocytes (*i.e.* SSTR-1, SSTR-2, and SSTR-4) [31], [35]. In our previous work, we reported that somatostatin is an allosteric modulator of IDE enzymatic activity on a fluorogenic Aβ-peptide [17]. Here, we show that somatostatin addition to the culture medium of BV-2 cells rapidly affects the amyloid β-peptide (1–40) extracellular concentration: after 1 h of stimulation (a time interval over which there is no accumulation of newly synthesized IDE), we observe a negative correlation between Aβ concentration and sst concentration. The lack of similar evidence in supernatants from IDE-silenced cells suggests that the decrease in Aβ levels is fully attributable to the modulation of IDE activity secreted before sst administration. As a whole, the reported data indicate that somatostatin regulates IDE expression, secretion and catalytic activity in microglia. These results are intriguing, considering that a microglia pharmacological manipulation is thought to play a neuroprotective role at least in the early stages of AD, since these cells cluster around senile plaques promoting Aβ phagocytosis and degradation [3], [40], [43]. Therefore, a correlation can be envisaged between the regulation of IDE activity and the microglial immunological function during the development of AD, opening a new therapeutic scenario for the control of AD in the early phases.

## Supporting Information

Figure S1 **Modulation of IDE expression by somatostatin.** Rat Astrocytes were incubated with indicated concentrations of somatostatin. Western blot analysis of normalized lysis samples indicates that no detectable effect of IDE expression is observed (left panel). Densitometric analysis of IDE WB signals (right panel). The results presented are the means ± ES of three independent experiments in triplicate, n = 9.
(TIF)
