# Supplementary figures and images for: Somatostatin Modulates Insulin-Degrading-Enzyme Metabolism: Implications for the Regulation of Microglia Activity in AD
Source: PLoS One. 2012 Apr 3;7(4):e34376. doi: 10.1371/journal.pone.0034376 (PMC3317975; doi:10.1371/journal.pone.0034376)

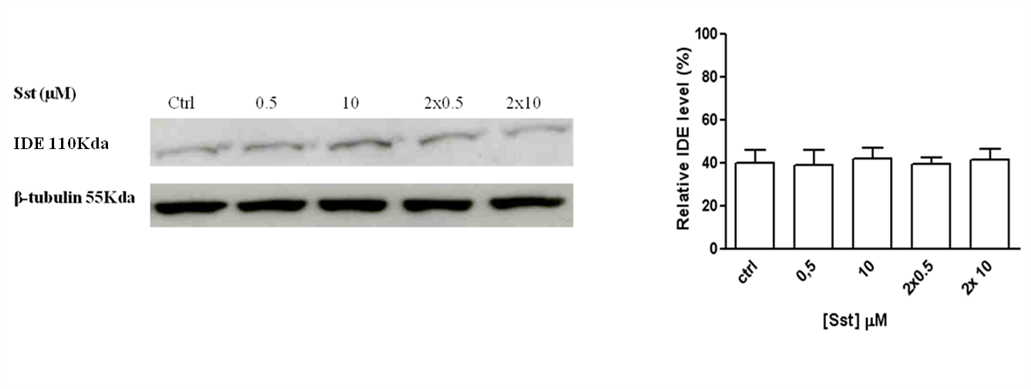

Supplement: Figure S1 — Modulation of IDE expression by somatostatin. Rat Astrocytes were incubated with indicated concentrations of somatostatin. Western blot analysis of normalized lysis samples indicates that no detectable effect of IDE expression is observed (left panel). Densitometric analysis of IDE WB signals (right panel). The results presented are the means ± ES of three independent experiments in triplicate, n = 9. (TIF) [file pone.0034376.s001.tif]
